# Comparative transcription analysis and toxin production of two fluoroquinolone-resistant mutants of *Clostridium perfringens*

**DOI:** 10.1186/1471-2180-13-50

**Published:** 2013-03-01

**Authors:** Sunny Park, Miseon Park, Fatemeh Rafii

**Affiliations:** 1 Division of Microbiology, National Center for Toxicological Research, US Food and Drug Administration, Jefferson, AR, USA; 2 Current Address; WO66 RM2467, 10903 New Hampshire Ave, Silver Spring, MD, 20993-0002, USA

**Keywords:** *Clostridium perfringens*, Fluoroquinolone, Resistance, Toxin, Virulence

## Abstract

**Background:**

Fluoroquinolone use has been listed as a risk factor for the emergence of virulent clinical strains of some bacteria. The aim of our study was to evaluate the effect of fluoroquinolone (gatifloxacin) resistance selection on differential gene expression, including the toxin genes involved in virulence, in two fluoroquinolone-resistant strains of *Clostridium perfringens* by comparison with their wild-type isogenic strains.

**Results:**

DNA microarray analyses were used to compare the gene transcription of two wild types, NCTR and ATCC 13124, with their gatifloxacin-resistant mutants, NCTR^R^ and 13124^R^. Transcription of a variety of genes involved in bacterial metabolism was either higher or lower in the mutants than in the wild types. Some genes, including genes for toxins and regulatory genes, were upregulated in NCTR^R^ and downregulated in 13124^R^. Transcription analysis by quantitative real-time PCR (qRT-PCR) confirmed the altered expression of many of the genes that were affected differently in the fluoroquinolone-resistant mutants and wild types. The levels of gene expression and enzyme production for the toxins phospholipase C, perfringolysin O, collagenase and clostripain had decreased in 13124^R^ and increased in NCTR^R^ in comparison with the wild types. After centrifugation, the cytotoxicity of the supernatants of NCTR^R^ and 13224^R^ cultures for mouse peritoneal macrophages confirmed the increased cytotoxicity of NCTR^R^ and the decreased cytotoxicity of 13124^R^ in comparison with the respective wild types. Fluoroquinolone resistance selection also affected cell shape and colony morphology in both strains.

**Conclusion:**

Our results indicate that gatifloxacin resistance selection was associated with altered gene expression in two *C. perfringens* strains and that the effect was strain-specific. This study clearly demonstrates that bacterial exposure to fluoroquinolones may affect virulence (toxin production) in addition to drug resistance.

## Background

*Clostridium perfringens* is commonly found in the gastrointestinal (GI) tract of humans, animals, soils, freshwater sediments and sewage. It can cause various diseases in humans, including food poisoning, antibiotic-associated diarrhea, sporadic diarrhea, internal abscesses, and gas gangrene and also various animal diseases [1-5]. *C. perfringens* strains all are prolific toxin producers and are classified based on their toxin formation. Various *C. perfringens* toxins denature cellular components of mammalian cells and are implicated in virulence and pathogenicity. Among these toxins are α-toxin (phospholipase C, PLC) and θ-toxin (perfringolysin O, PFO), which are essential for gas gangrene pathogenesis. Other toxins or hydrolytic enzymes may be involved in destruction of connective tissue or spread of bacteria in infected tissues [4,6,7]. *C. perfringens,* although a commensal, can cause life threatening infections and is implicated in inflammatory bowel diseases [8-10]. In a survey of *Clostridium* species bacteremia, in a Canadian hospital between 2000–2006, *C. perfringens* was shown to have caused 42% of the cases, which was more than any other *Clostridium* species [11]. It causes nearly a million cases of food borne illness each year in the United States [1]. Bacteria from the GI tract, including *C. perfringens*, may become resistant to fluoroquinolones used for treatment or prophylaxis of bacterial infections. Surveys of fluoroquinolone-resistant-anaerobes found ciprofloxacin-resistant *C. perfringens* as early as 1992 among clinical isolates [12]. Although similar surveys have not been conducted in recent years, Gionchetti et al. [10] showed that treatment of patients with chronic treatment-resistant pouchitis with 1 g of ciprofloxacin for 15 days did not result in a statistically significant reduction in *C. perfringens*. One reason for fluoroquinolone resistance development is mutation in the fluoroquinolone target genes, gyrase (*gyrA* and *gyrB*) and topoisomerase IV (*parC and parE*) [13]. Because fluoroquinolones are DNA-damaging agents, they may also induce the SOS response [14-16] that results in expression of DNA repair genes, which may lead to phenotypic changes in fluoroquinolone-resistant strains [17-20]. Excessive use of fluoroquinolones has been attributed to the emergence of virulent strains of bacteria [21-24]. *Clostridium difficile* strain NAP1/027, which emerged in 2002 in Canada and the USA, now has spread to most parts of Europe [22]. In a gut model, higher rates of spore germination and levels of toxin production were observed in two ribotypes of *C. difficile* that were exposed to three different fluoroquinolones [24]. Wide dissemination of virulent fluoroquinolone-resistant strains of *Escherichia coli* has been reported in East Asia [25]. Other reports, sometimes conflicting, show either increased or decreased virulence in fluoroquinolone-resistant clinical isolates of bacteria [26-28]. Previously we showed that different *C. perfringens* strains rapidly developed resistance, even to high potency fluoroquinolones, and that resistant strains had various mutations in the fluoroquinolone target genes [29]. In addition, the production of some enzymes was altered in some resistant mutants [30,31]. One gatifloxacin-resistant strain, NCTR, had increased levels of α-toxin (phospholipase C, PLC) and θ-toxin (perfringolysin O, PFO) [30]. These results point to global changes in the expression of various genes in gatifloxacin- resistant strains and to the need for further study. In this study, we have used genomic analysis (microarray and QRT-PCR) to compare the changes in gene expression in two gatifloxacin-resistant strains of *C. perfringens* following fluoroquinolone resistance selection, and have compared the toxin production and cytotoxicity of the strains. Strain NCTR was selected because of enhanced production of PLC and PFO by its gatifloxacin resistant mutant and was compared with strain ATCC 13124, which is a gangrene isolate whose genomic sequence is known, and its gatifloxacin resistant mutant 13124^R^ has the same mutation in *gyrA* as NCTR^R^.

## Methods

### Growth of bacterial strains

Wild types and gatifloxacin-resistant mutants of *C. perfringens* strains ATCC 13124 and NCTR [29] were used in this study. The development of these mutants (Gat-13124-10 and Gat-NCTR- 10) was described previously [29]. Both mutants have stable mutations in target genes and will be referred to as 13124^R^ and NCTR^R^ in this study. Both mutants had a mutation in *gyrA* (G81C, D87Y), 13124^R^ had mutation in *gyrB* (A431S) and *parC* (S89I), and NCTR^R^ had a mutation in *parE* (E486K). The bacteria were grown anaerobically under an atmosphere of 85% N_2_, 10% CO_2_, and 5% H_2_ at 37°C in brain heart infusion (BHI) broth (Remel, Lenexa, KS) with vitamin K (1 μg/ml), hemin (5 μg/ml), and L-cysteine (5 μg/ml) (Sigma Chemical Co., St. Louis, MO) [29]. No antibiotics were added.

### Preparation of RNA

Early exponential (2.5-3.0 h) growth phase cultures of all four strains, grown in BHI under identical anaerobic conditions, were used to isolate RNA for microarrays. Cells from 100-ml cultures were harvested by centrifugation (15,000 × *g*, 10 min, 4**°**C), washed with 10 mM Tris and 1 mM EDTA (pH 8.0), and suspended in 1 ml of buffer containing 10 mg/ml of lysozyme (Sigma). The mixtures were incubated for 10 min at room temperature and centrifuged (15,000 × *g*, 10 min, 4**°**C). The samples were suspended in 0.5 ml TE (10 mM Tris, 1 mM EDTA) and mixed with 5 ml of RNA-Bee isolation reagent from TEL-TEST, Inc. (Friendship, TX). After addition of 1 ml chloroform to the mixture, the samples were incubated on ice for 30 min and centrifuged (15,000 × *g*, 30 min, 4**°**C). The clear phases were harvested, added to an equal volume of isopropanol and centrifuged to pellet the RNA. The RNA was further purified using an RNeasy^R^ Mini Kit (50) from QIAGEN, Inc. (Valencia, CA), according to the instructions provided with the kit. After RNA extraction and purification, contaminating DNA was removed using 10 U of RNase-free DNase 1 (Boehringer Mannheim, Ingelheim, Germany). The quantity and quality of total RNA was determined using a Nanodrop ND-1000 spectrophotometer (NanoDrop Technology, Wilmington, DE). RNA purification steps for real-time PCR (qRT- PCR) were essentially the same. The RNA was stored at −80**°**C and used within a week to avoid degradation of RNA. RNA was extracted from three different cultures of each strain for microarray analysis and qRT-PCR.

### Probe design for microarrays

The probes were designed by Biodiscovery LLC, Ann Arbor, MI (http://www.mycroarray.com/) from the sequences of *C. perfringens* strains 13 (CPE) and ATCC 13124 (CPF in http://www.ncbi.nlm.nih.gov), using OligoArray v 3.1 (http://berry.engin.umich.edu). The designs of microarrays were submitted to MIAMExpress and can be accessed at the following links: for strain 13124, [http://www.ebi.ac.uk/arrayexpress/arrays/A-MEXP-2008], and for strain NCTR, at [http://www.ebi.ac.uk/arrayexpress/arrays/A-MEXP-2027].

### Microarray hybridization

The microarrays were hybridized by Biodiscovery LLC to fluor-labeled RNA at 60**°**C for at least 16 h in 2-gasket slides and commercial hybridization chambers (Agilent, Santa Clara, CA) while being rotated (~4 rpm) in a hybridization incubator. The hybridization solution contained 6 × SSPE (1 M NaCl, 50 mM NaH_2_PO_4_, 50 mM Na_2_HPO_4_, 3 mM EDTA, pH 6.7), 1 μg/μl acetylated BSA, 1 μg/μl herring sperm DNA (Promega, Madison,WI), 0.01% Tween 20 (Sigma) and 10 μg template RNA per array. The hybridized arrays were washed twice in 6 × SSPE for 5 min at 60**°**C, once in 1 × SSPE for 5 min at 20**°**C, and once in 0.25 × SSPE at 20**°**C for 1 min, and then were spun dry in a microarray high-speed centrifuge (ArrayIT, model MHC). The arrays were scanned in an Axon 4000B scanner (Molecular Devices Sunnyvale, California), controlled by GenePixPro software (v 6.1.0.4). The resulting images were quantified with the same software and the results were archived in the *gpr* file format. The mean expression of each gene for the mutant was divided by the mean expression of the same gene for the wild type. Those genes for which the values were ≥ 1.5 were considered upregulated in the mutant, and the genes for which this value was ≤0.6 were considered downregulated in the mutant. The genes that were upregulated or downregulated were selected for further RT-PCR analysis.

### Quantitative real-time PCR (qRT-PCR)

Primers used for qRT-PCR are listed in Additional file 1. The genes that were upregulated in one mutant and downregulated in the other mutant, in comparison with their respective wild types, by microarray analysis were selected to design primers. Some genes involved in regulation of transcription were also selected. The sequence of *C. perfringens* ATCC 13124 (http://www.ncbi.nlm.nih.gov/nuccore/CP000246.1) was used to design primers that generated PCR amplicons of 100–150 bp in length via the default setting of “Primer 3 Input software” (http://frodo.wi.mit.edu/primer3). For cDNA template synthesis, SuperScriptTM III First-Strand Synthesis SuperMix (Invitrogen, Carlsbad, CA) was used. For qRT-PCR, SYBR^®^ GreenER^TM^ qPCR SuperMix (Invitrogen) was used. The reaction mixtures were prepared on ice according to the manufacturer’s instructions. Each reaction contained 2 × Express SYBR Green ER qRT-PCR universal mix, 25, 2.5, or 0.25 ng of the cDNA template, and 2 μM each of the forward and reverse primers. The amplification was performed using a CFX96 Real-Time PCR detection system (Bio-Rad, Hercules, CA) and the following protocol: 50**°**C for 10 min, 95°C for 8.5 min to inactivate uracil DNA glycosylase and activate DNA polymerase, followed by 40 cycles of 95°C for 15 s and 60°C for 1 min to amplify cDNA. Melting curves were monitored at 65-95°C (1°C per 5 s) to detect any nonspecific amplification. Either 25, 2.5, or 0.25 ng of each 16S rRNA gene was amplified as a reference RNA of equivalent size for normalization [32]. Reaction mixtures without reverse transcriptase, for detecting genomic DNA contamination, and reaction mixtures without templates, for detecting nucleic acid contamination of reagents and tubes, were included as controls. Each PCR reaction was run in triplicate for each type of RNA isolated from three different cultures of each wild type or mutant. The relative level of mRNA expression was calculated by the 2^-ΔΔC^T method according to Real-Time PCR Application Guide (Additional file 2).

### Detection of phospholipase C (PLC) and perfringolysin O (PFO)

PLC and PFO activities were measured according to the methods previously described [7,30,33]. The hemoglobin release from red blood cells in the presence of perfringolysin buffer was measured to detect perfringolysin O (PFO) according to the method of O'Brien and Melville [33]. The increase in turbidity of lecithin in egg yolk emulsion or the release of nitrophenol from *O*-(4-nitrophenyl-phosphoryl) choline as the result of hydrolysis by PLC was used to measure phospholipase C (PLC) activity [7,30].

### Collagenase assay

The amounts of collagenase in the mutants and wild types were calculated by the method of Awad et al. [34] by measuring the amount of dye released from Azo Dye Impregnated Collagen (azocoll) (Sigma). Azocoll powder was washed and resuspended in 0.2 M of borate buffer (pH 7.2) containing 0.15 M NaCl, 20 μM ZnCl_2_ and 5 mM CaCl_2_ to a final concentration of 5 mg azocoll per ml. Next, 100 μl of the filter-sterilized supernatants of centrifuged wild types and mutants were added to 400 μl of azocoll solution and the mixtures were incubated for 2 h at 37**°**C. Following centrifugation at 16,100 × *g*, the released dye was measured by the absorbance at 550 nm.

### Assay for clostripain

A clostripain substrate, *N*-carbobenzoxy-L-arginine *p*-nitroanilide (Z-Arg-pNA) (Bachem Americas, Torrance, CA), was used for measuring the amounts of clostripain in the supernatants of wild types and mutants [35]. The filter-sterilized supernatant from each centrifuged strain was incubated overnight at 4°C in a buffer containing dithiothreitol to reduce the thiol group of the cysteine residues of clostripain. Next, 20 μl of the sample was added to the 300 μl buffer containing 2 mM CaCl_2_ and 260 mM of Z-Arg-pNA. The kinetics software of the spectrophotometer was programmed to measure the absorbance at 410 nm every min for 30 min. The amount of cleavage of Z-Arg-pNA was measured and the enzyme units were calculated. One unit was defined as the amount of enzyme that hydrolyzed 1.0 μmol of Z-Arg-pNA per min [35].

### Detection of sialidase

Sialidase activity was measured in filter-sterilized supernatants of centrifuged cultures of mutants and wild types, using 4 mM 5-bromo-4-chloro-3-indolyl-α-D-*N*-acetylneuraminic acid, sodium salt [36]. The assay reaction was performed in 96-well plates by addition of the supernatant to wells containing the substrates, according to a procedure recommended by Sigma for measuring recombinant *C. perfringens* neuraminidase. The kinetics software was programmed to measure the absorbance at 595 nm.

### Hyaluronidase detection

The amounts of hyaluronidase in the filter-sterilized supernatants of centfifuged wild types and mutants were quantified by measuring the degradation of hyaluronic acid. Bovine hyaluronic acid (Sigma) was dissolved in acetate buffer (200 mM sodium acetate, 150 mM NaCl, pH 6.0) to a final concentration of 1 mg/ml. 100 μl of the hyaluronic acid solution was incubated with 400 μl of the filter-sterilized supernatants of the wild types and mutants for 30 min at 37**°**C. One ml of a solution containing 2% NaOH and 2.5% cetramide (cetyltrimethylammonium bromide, Sigma) was added to the reaction mixture. The turbidity of the insoluble complex formed between cetramide and hyaluronic acid was measured at 400 nm [37]. The reduction in turbidity, reflecting the decrease in hyaluronic acid because of the activity of hyaluronidase, was calculated by comparing the turbidities of samples containing the supernatant of each culture with controls containing BHI alone. The enzyme assays for all the enzymes were performed three times from three different cultures of each strains.

### Cytotoxicity of *C. perfringens* supernatants for macrophages

Macrophages were obtained from C57BL/6 male mice, 4–6 weeks old, which had ad libitum access to food and water. The maintenance, handling and sacrifice of mice were according to procedures approved by the NCTR Institutional Animal Care and Use Committee. Resident mouse peritoneal macrophages were harvested by peritoneal lavage, using 4 ml of supplemented DMEM medium, containing 5% heat-inactivated fetal bovine serum, 100 μg/ml streptomycin sulfate, 100 units/ml penicillin G, 110 mg/L sodium pyruvate, and 2 mM glutamine. Red blood cells were removed by hypotonic lysis. The peritoneal exudate cells were washed once with DMEM, plated and incubated at 37°C in a humidified atmosphere of 5% CO_2_[33]. Floating cells were removed and the macrophages were incubated in DMEM, containing 10% BHI or filter-sterilized supernatants of overnight cultures of wild types and mutants, for 18 h at 37°C in a CO_2_ incubator. A CytoTox 96^®^ Non-Radioactive Cytotoxicity Assay Kit (Promega) was used to measure the toxicity of the mutants and wild type cultures for macrophages. The cytotoxicity of each absorbance unit of the cells of different strains was calculated by the amount of lactate dehydrogenase (LDH) released from the macrophages. The differences in cytotoxicity due to the mutants and wild types were assessed using Student’s *t*-test.

### Morphological examination

Colony morphology of the strains was compared after overnight growth on BHI plates. For cellular morphology, log phase grown cells were Gram stained and examined under the light microscope.

### DNA sequencing

Several regulatory and toxin genes and enzymes from wild types and mutants were amplified and sequenced as previously described [29].

## Results

### Transcriptional analysis by DNA microarray

Using the genome sequences of *C. perfringens* strain 13 and strain ATCC 13124, microarray probes were designed for genome-wide transcriptional analysis of two fluoroquinolone-resistant *C. perfringens* strains, NCTR^R^ and 13124^R^, and their wild types. Microarray analysis showed that a variety of genes were upregulated (≥ 1.5 fold) or downregulated (≤ 1.5 fold) in the fluoroquinolone-resistant strains. The altered genes with known functions that were affected in both strains as the results of fluoroquinolone resistance selection are grouped in Tables 1, 2, 3 according to the classification used by the Institute for Genomic Research (http://www.jcvi.org/). In addition, the microarray detected alterations of many genes, for which the function is not known, which are listed as hypothetical proteins in the GenBank. Some of these were upregulated manyfold in both resistant strains, especially in 13124^R^. The genes that were differentially affected in the resistant strains are shown in Table 1. Many of these genes were generally upregulated in NCTR^R^ and downregulated in 13124^R^. The common genes that were upregulated in one or both mutants are in Table 2 and those that were downregulated in both are in Table 3. Some genes involved in amino acid biosynthesis, protein synthesis, fatty acid synthesis, and phospholipid metabolism were mostly upregulated in 13124^R^. Some genes for putative membrane proteins were upregulated in either one (Table 1) or both mutants (Table 2). The ATP synthase and potassium transporter genes were upregulated in both mutants (Table 2). Some of the genes involved in purine, pyrimidine, nucleotide, and nucleoside transport and metabolism were upregulated in both mutants and some were downregulated in both mutants (Tables 2 and 3). Several transcriptional regulators, transporters and kinases also were downregulated in one or both mutants (Tables 1 and 3). Resistance selection also affected the expression of genes involved in virulence (phospholipase C, perfringolysin O, collagenase, hemolysin III and α-clostripain). Surprisingly, these genes were upregulated in strain NCTR^R^ and downregulated in strain 13124^R^.

**Table 1:** **Microarray and qRT-PCR analysis of the genes that were differentially affected in the gatifloxacin resistant mutants, NCTR**^**R **^**and 13124**^**R**^

**Gene ID and name**	**Function**	**Microarray**		**qRT-PCR**	
		**mt/wt**		**mt/wt**	
		**NCTR**	**ATCC 13124**	**NCTR**	**ATCC 13124**
**Cell envelope**					
CPE1089 CPF_1345	putative membrane protein	4.3	−2.1	7.3	−2.8
CPE0162 CPF_0155 (*pfoR*)	putative membrane protein	2.6	−4.0	3.3	−3.5
CPE0251 CPF_0244	putative lipoprotein	5.0	−2.4	2.0	−3.5
CPE0278 CPF_0274 (*sagA*)	sagA protein	1.1	−2.4	4.7	−2.6
CPE0714 CPF_0710	putative monogalactosyl-diacylglycerol synthase	2.4	−2.4	7.6	6.3
**Cellular processes**					
CPE0036 CPF_0042 (*plc*)	phospholipase C	4.8	−6.8	1.9	−3.3
CPE0846 CPF_0840 (*cloS1*)	α-clostripain	17.3	−15.6	8.3	−1143
CPE1474 CPF_1725 (*hlyC*)	hemolysin III	3.2	−1.8	15.1	−2.6
CPE0163 CPF_0156 (*pfoA*)	perfringolysin O	3.6	−71.4	6.4	−462
CPE0782 CPF_0784 (*ahpC*)	alkyl hydroperoxide reductase-C subunit	10.3	−2.6	13.4	−12.6
CPE1092 CPF_1348 (*pac*)	choloylglycine hydrolase family protein	1.7	−2.5	25.7	−1.7
**Energy metabolism**					
CPE0778 CPF_0780	oxidoreductase, FDA-binding	4.8	−2.8	85	2.6
CPE1299 CPF_1505 (*eno*)	enolase	3.5	−1.6	11.9	−1.9
CPE2058 CPF_2315 (*gadB*)	glutamate decarboxylase	31.9	−3.5	20.0	−3.4
CPE2437 CPF_2747 (*nrdH*)	glutaredoxin-like protein, YruB-family	3.8	−2.5	4.8	−11.0
CPE2551 CPF_2875 (*glpA*)	probable glycerol-3-phosphate dehydrogenase	0.8	−2.5	1.3	−0.1
**Purines, pyrimidines, nucleotides, and nucleosides**					
CPE2276 CPF_2558 (*guaB*)	inosine-5’-monophosphate dehydrogenase	9.2	−3.6	30.3	−1.5
CPE2622 CPF_2958 (*purA*)	adenylosuccinate synthetase	4.3	−1.9	14.8	−0.8
**Protein fate**					
CPE0173 CPF_0166 (*colA*)	collagenase	9.9	−4.7	8.5	−2.7
CPE2323 CPF_2632 (*pepF)*	probable oligoendopeptidase F	2.7	-2.0	11.6	4.3
CPE1205 CPF_1002 (*abgB*)	amidohydrolase family protein	1.9	−4.3	67.4	−1.6
**Regulatory functions**					
CPE0073 CPF_0069	transcription antiterminator	2.1	−5.0	1.9	−2.6
CPE0759 CPF_0753	putative regulatory protein	1.5	−5.4	3.3	0.6
CPE1533 CPF_1784 (*scrR*)	sucrose operon repressor	1.7	−2.8	132	−1.5
CPE2035 CPF_2292 (*hrcA*)	heat-inducible transcription repressor HrcA	2.3	−2.9	9.5	5.5
CPE2363 CPF_2673	two-component sensor histidine kinase	2.1	−3.0	16.1	2.7
**Transport and binding proteins**					
CPE1240 CPF_1450 (*mgtE*)	magnesium transporter	8.6	−1.7	5.2	−2.6
CPE1300 CPF_1507 (*gadC*)	glutamate:γ-aminobutyrate antiporter family protein	9.6	−2.7	17.1	−7.3
CPE1505 CPF_1756 (*uraA*)	uracil transporter	3.8	−2.7	3.9	−4.6
CPE0075 CPF_0070	N-acetyl glucosamine-specific	1.4	−14.3	1 .8	ND
CPE0707 CPF_0703	ABC transporter, ATP-binding protein	1.5	−3.2	5.2	2.9
CPE0761 CPF_0756 (*gltP*)	proton/sodium-glutamate symporter	1.5	−4.2	4.6	0.9
CPE1371 CPF_1621	sodium:neurotransmitter symporter family protein	1.8	−4.0	15.2	2.7
CPE2084 CPF_2341 (*modB*)	molybdate ABC transporter, permease protein	1.8	−2.5	10.8	2.0
CPE2343 CPF_2652 (*malE*)	putative maltose/maltodextrin ABC transporter	2.9	1.3	3.8	−2.1
**Unknown functions**					
CPE0183 CPF_0176	nitroreductase family protein	1.0	−4.8	2.9	−1.1
CPE1172 CPF_1375	haloacid dehalogenase	2.1	−2.4	20.6	−1.7
CPE1784 CPF_2038 (*nifU*)	NifU family protein	1.3	−2.5	6.4	−1.5
CPE2448 CPF_2758	PSP1 domain-containing protein	1.0	−2.4	5.5	−1.9

All of the data are the means of three different experiments.

**Table 2:** **Microarray analysis of the genes that were upregulated in one or both gatifloxacin-resistant mutants, 13124**^**R **^**and NCTR**^**R**^

**Gene ID and name**		**Function/Similarity**	**Microarray (mt/wt)**	
			**NCTR**	**ATCC 13124**
**Amino acid biosynthesis**				
CPE1520	CPF_1772 (*ilvE*)	branched-chain amino acid aminotransferase	1.1	2.6
CPE1905	CPF_2161 (*dapA*)	dihydrodipicolinate synthase	1.0	1.9
**Cell envelope**				
CPE0492	CPF_0465	capsular polysaccharide biosynthesis protein	6.5	1.9
CPE0495	CPF_0468	UDP-glucose/GDP-mannose dehydrogenase family	3.5	2.4
CPE2059	CPF_2316	putative membrane protein	7.1	3.2
CPE2079	CPF_2336	putative membrane protein	14.2	2.1
CPE0785	CPF_0787	putative membrane protein	2.3	2.1
**Energy metabolism**				
CPE2186	CPF_2451 (*atpE*)	ATP synthase epsilon subunit	3.3	2.9
CPE2187	CPF_2452 (*atpB*)	ATP synthase beta subunit	3.6	2.2
CPE2189	CPF_2454 (*atpA*)	ATP synthase alpha subunit	4.2	2.4
CPE2190	CPF_2455 (*atpH*)	ATP synthase delta subunit	1.9	2.1
CPE2191	CPF_2456 (*atpF*)	ATP synthase B chain	2.2	2.3
CPE2192	CPF_2457 (*atpL*)	ATP synthase C chain	3.6	2.3
**Fatty acid and phospholipid metabolism**				
CPE1068	CPF_1324 (*fabH*)	3-oxoacyl-(acyl-carrier-protein) synthase III	2.2	4.7
CPE1069	CPF_1325 (*fabD*)	malonyl CoA-acyl carrier protein transacylase	1.1	3.6
CPE1071	CPF_1327 (*fabF*)	3-oxoacyl-(acyl-carrier-protein) synthase II	1.3	3.8
CPE1072	CPF_1328 (*accB*)	acetyl-CoA carboxylase, biotin carboxyl carrier	0.9	4.0
CPE1073	CPF_1329 (*fabZ*)	beta-hydroxyacyl-(acyl-carrier-protein) dehydratase FabZ	1.0	4.5
CPE1074	CPF_1330 (*accC*)	acetyl-CoA carboxylase, biotin carboxylase	1.7	4.9
CPE1075	CPF_1331 (*accD*)	acetyl-CoA carboxylase, carboxyl transferase, beta subunit	3.4	5.0
CPE1076	CPF_1332 (*accA*)	acetyl-CoA carboxylase, carboxyl transferase, alpha subunit	1.9	4.6
**Protein synthesis**				
CPE1697	CPF_1951 (*frr*)	ribosome recycling factor	1.1	2.0
CPE2441	CPF_2720	ribosomal protein L7AE family	1.1	2.6
CPE2660	CPF_2997 (*rpmH*)	ribosomal protein L34	1.4	2.0
**Purine, pyrimidine, nucleotides, and nucleosides**				
CPE1050	CPF_1305 (*mtnH*)	5-methylthioadenosine/S-adenosylhomocysteine nuclosidase	3.2	2.6
CPE2162	CPF_2418 (*cpdC*)	2`,3`-cyclic-nucleotide 2`-phosphodiesterase	3.4	1.6
**Transport and binding proteins**				
CPE0977	CPF_1235	potassium transporter	7.1	2.9
**Unknown functions**				
CPE2601	CPF_2928	conserved hypothetical protein	6.7	58.0

All of the data are the means of three different experiments.

**Table 3:** **Microarray analysis of the genes that were downregulated in both gatifloxacin-resistant strains, 13124**^**R **^**and NCTR**^**R**^

**Gene ID (name)**		**Function/Similarity**	**Microarray (mt/wt)**	
			**NCTR**	**ATCC 13124**
**Biosynthesis of cofactors, prosthetic groups, and carriers**				
CPE1085	CPF_1341 (*ispH*)	4-hydroxy-3-methylbut-2-enyl diphosphate reductase	−2.4	−2.2
**Energy metabolism**				
CPE0292	CPF_0288	carbohydrate kinase family protein	−3.1	−2.5
CPE1185	CPF_1389 (*pfk*)	6-phosphofructokinase	−1.7	−2.7
CPE0585	CPF_0565 (*fruB*)	fructose-1-phosphate kinase	−5.2	−2.3
CPE0692	CPF_0684	transaldolase	−2.8	−2.3
CPE0725	CPF_0721 (*nanI*) ^*^	exo-alpha-sialidase	−3.5	1.5
CPE0894	CPF_0887 (*eutP*)	ethanolamine utilization protein, EutP	−1.9	−2.0
CPE2348	CPF_2657 (*ptb*)	phosphate butyryltransferase	−2.3	−1.6
**Purine, pyrimidine, nucleotides, and nucleosides**				
CPE1398	CPF_1652 (*deoD*)	purine nucleoside phosphorylase	−1.7	−3.4
**Regulatory functions**				
CPE0586	CPF_0566 (*fruR*)	transcriptional regulator, DeoR family	−3.6	−2.6
CPE0631	CPF_0612	probable PBP5 synthesis regulator protein	−2	−2.5
CPE1077	CPF_1333	transcriptional regulator, PadR family	−3.1	−3.2
CPE2510	CPF_2833	transcriptional regulator, PadR family	−2.6	−2.7
CPE1305	CPF_1512	probable transcriptional regulator	−2	−1.6
**Transport and binding proteins**				
CPE0600	CPF_0581	amino acid ABC transporter	−4.8	−3.4
CPE1534	CPF_1785	PTS system, sucrose-specific IIBC component	−3.1	−14.3
CPE2345	CPF_2654	putative maltose/maltodextrin ABC transporter	−2.0	−1.8
**Unknown functions**				
CPE2509	CPF_2832	degV family protein	−3.6	−3.3
CPE1171	CPF_1374	mutator mutT protein homolog	−6.4	−2.0
CPE2592	CPF_2917	phnA family protein	−2. 8	−2.0

^*^ Decrease in the expression of *nanI* in NCTR^R^ and increase of its expression in 13124^R^ was confirmed by qRT-PCR.

All of the data are the means of three different experiments.

### Validation of DNA microarray data by qRT-PCR

To verify that fluoroquinolone resistance selection indeed had different effects on the expression of some of the genes in *C. perfringens*, the transcription of the genes that were generally upregulated or unchanged in NCTR^R^ and downregulated in 13124^R^ was measured by qRT-PCR (Table 1). Real-time PCR verified the upregulation of all of the genes that were tested in NCTR^R^ and downregulation of a majority of the genes that were downregulated in 13124^R^. qRT-PCR was also performed on the genes that are reported to have regulatory functions (Table 4). *virR*, *virS*, *vrr*, *virX* and others were all upregulated in NCTR^R^ by at least twofold. In strain 13124^R^, *virX* was downregulated more than twofold, but *vrr* also was substantially downregulated. Among the genes whose expression was altered by fluoroquinolone resistance selection were phospholipase C (PLC), perfringolysin O (PFO), α-clostripain, hemolysin III, and collagenase. Both microarray analysis and qRT-PCR showed upregulation of these genes in NCTR^R^ and downregulation in 13124^R^. Both microarray and qRT-PCR showed downregulation of the sialidase gene, *nanI*, in NCTR^R^ and upregulation of this gene in 13124^R^.

**Table 4:** **Results of qRT-PCR for the *C. perfringens *regulatory genes in the wild types and mutants**

**Gene ID and name**		**Regulatory function**	**qRT-PCR fold**	
			**change (mt/wt)**	
			**NCTR**	**ATCC13124**
CPE_1501	CPF_1752 (*virR*)	DNA binding response regulator, VirR	7.4	1.3
CPE_1500	CPF_1751 (*virS*)	sensor histidine kinase, VirS	9.7	0.3
CPE_0646	CPF_0627 (*virX*)	conserved hypothetical protein	2.2	−3.0
CPE_0957	CPF_1204 (*vrr*)	VR-RNA	2.0	−158.5
CPE_1701	CPF_1955 (*codY*)	GTP-sensing transcriptional pleiotropic repressor CodY	6.9	−1.8
CPE_0073	CPF_0069	Transcription antiterminator	1.5	−116.5
CPE_0642	CPF_0623 (*RevR*)	DNA binding response regulator	2	−2

### Toxin production in the mutants and wild types

**Figure 1:**
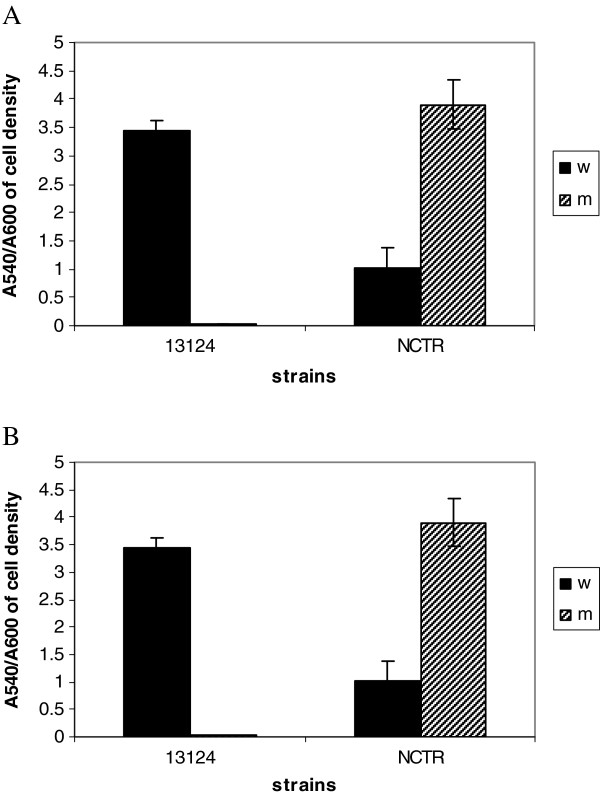
**Comparison of phospholipase C (A) and perfringolysin O (B) activities of the wild type strains of *C. perfringens*, ATCC 13124 and NCTR, with their respective mutants, 13124**^**R **^**and NCTR**^**R**^**.** W: wild type, M: mutant.

**Figure 2:**
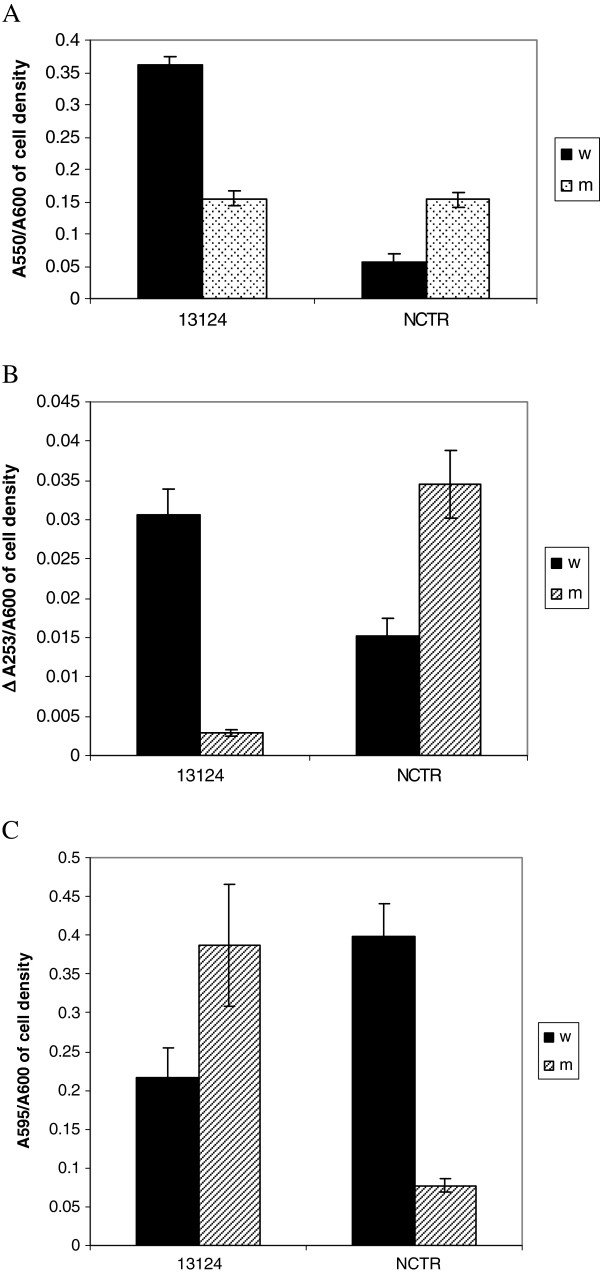
**Comparison of collagenase (A), clostripain (B) and sialidase (C) activities of the wild type strains of *C. perfringens, *ATCC 13124 and NCTR, with their respective mutants, 13124**^**R **^**and NCTR**^**R**^**.** W: wild type, M: mutant.

The quantities of several enzymes that are implicated in bacterial virulence were measured for each absorbance unit of cells of wild types and mutants of both strains (Figures 1 and 2). The production of phospholipase C (PLC), perfringolysin O (PFO), collagenase, clostripain, and sialidase were all affected in the resistant mutant. Strain 13124^R^ produced less PLC and PFO than the wild type. In contrast, as previously reported [30], the production of both enzymes increased in NCTR^R^. Collagenase and clostripain production also were similarly affected by fluoroquinolone resistance selection, but the most dramatic effect was for perfringolysin O (PFO) in ATCC 13214, which was totally inhibited in 13124^R^. However, sialidase had increased in 13124^R^ but decreased in NCTR^R^. Hyaluronidase was not significantly affected. The alterations in the production of PLC, PFO, collagenase and clostripain in both strains reflected the alteration of gene expression for these enzymes, as shown by microarray and qRT-PCR.

### Cytotoxic effects on mouse peritoneal macrophages

**Figure 3:**
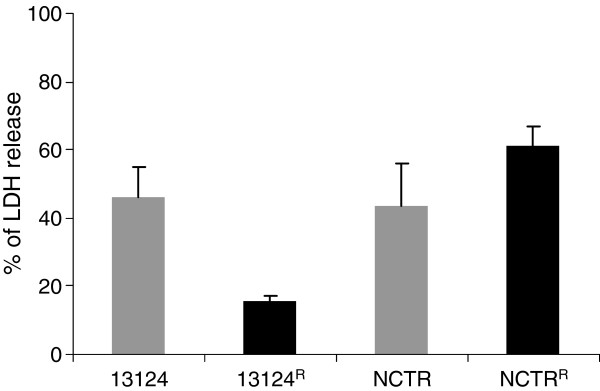
**Comparison of cytotoxicity of two gatifloxacin-resistant *C. perfringens *mutant strains, 13124**^**R **^**and NCTR**^**R**^**, with their wild type parents, strains ATCC 13124 and NCTR, for peritoneal macrophages, as measured by LDH (lactate dehydrogenase) released.**

To investigate if the changes in the expression levels of toxin genes in the fluoroquinolone resistant mutants affected cytotoxicity for phagocytes, cytotoxicity assays were performed by incubating mouse peritoneal macrophages with cell-free filtrates of the centrifuged bacterial cultures. The levels of cytotoxicity were compared by measuring the amount of lactate dehydrogenase (LDH) released from the lysed macrophages. The relative cytotoxicity was about threefold lower (*P*= 0.0131) in 13124^R^ than in ATCC 13124 (Figure 3). The supernatant of NCTR^R^ showed about 1.4-fold higher cytotoxicity than that of NCTR. Microscopic observation also indicated that macrophages treated with bacterial culture media from ATCC 13124 and NCTR^R^ were rounded off and detached from the surface (Additional file 3).

### Morphological examination

Gram staining of log phase cultures showed that gatifloxacin resistance selection affected the shape of cells (Additional file 4). As expected, the Gram reaction was positive for both wild types and their mutants. The resistant mutants were more elongated than the wild types but the amounts of elongation and differences in cell shape were much more pronounced for the NCTR/NCTR^R^ strain pair than for the ATCC 13214/13124^R^ strain pair. Fluoroquinolone resistance selection also affected the colony morphology of the resistant strains. The colony size of NCTR^R^ was bigger than that of the wild type, and the colony size of 13124^R^ was smaller than that of the wild type (Additional file 4).

## Discussion

The use of fluoroquinolones has been listed as a risk factor for the emergence of virulent antibiotic-resistant strains of some bacteria [21-23]. We studied the effect of fluoroquinolone resistance selection on the global transcriptional response in gatifloxacin-resistant *C. perfringens* strains 13124^R^ and NCTR^R^ by microarray analysis. The fluoroquinolone resistance selection resulted in alteration of transcription levels of a significant number of genes involved in almost every aspect of metabolism in the resistant mutants of both strains in comparison with their wild types. Many genes with similar functions were either upregulated or downregulated in the resistant mutants. However, some genes that were downregulated in 13124^R^ were upregulated in NCTR^R^. qRT-PCR analysis confirmed that the transcription of these genes, which included toxin genes for phospholipase C (PLC), perfringolysin O (PFO), collagenase and clostripain, were affected differently in the two mutants. Similarly, the production of these enzymes and the toxicity of the culture supernatants decreased in 13124^R^ and increased in NCTR^R^. It appears that gatifloxacin resistance selection resulted in alteration of global gene transcription in *C. perfringens* and that the effect was strain-specific.

The changes in the levels of global gene expression due to the response to fluoroquinolone exposure may be governed by complex regulatory processes. Both resistant strains harbored some common and some unique mutations in fluoroquinolone target genes. These enzymes are involved in the DNA supercoiling process that plays an essential role during gene transcription [38,39]. Although neither of the resistant strains was a clinical isolate, some of the mutations found in the resistant strains were the same as those found in fluoroquinolone-resistant mutants of *E. coli* obtained from clinical samples, which were also the same as those found in fluoroquinolone-resistant mutants of *E. coli* generated in the laboratory [29,40].

The expression of a number of genes is affected by supercoiling [19] and aberrant expression of these genes occurs when DNA supercoiling has been altered by gyrase mutation(s). Alleles of *gyrA* that reduce DNA supercoiling have been shown to generate metabolic defects and reduce fitness of gyrase mutant strains [38,41]. Furthermore, because fluoroquinolones are DNA-damaging agents, in addition to inducing mutation in target genes, changing DNA superhelicity, they may also induce the expression of DNA repair genes via the SOS response, which may lead to phenotypic changes [15,17-20]. Cirz et al. [15] characterized the global transcription response of *S. aureus* to ciprofloxacin and, among other changes, found induction of the SOS response, upregulation of the TCA cycle and downregulation of α-hemolysin and a leukocidin family toxin. The positive regulators of transcriptional responses for stress and toxin genes were also downregulated [15]. In *C. perfringens*, although the expression of several virulence genes decreased in one resistant mutant (13124^R^), it increased in another (NCTR^R^). The transcription of various genes, including toxin genes, is regulated by *virR* and *virS*[32,42,43]. VirS is a sensor histidine kinase, which autophosphorylates in response to extracellular signals, and VirR is a response regulator [32,42,43]. These two genes, along with *vrr* (which is an RNA regulator virR-RNA), are implicated in controlling gene transcription [44] and were upregulated in NCTR^R^. In 13124^R^, transcription of *VirR* did not change, and *virS* and *vrr* were downregulated. The gene *vrr* is directly regulated by *VirR/VirS*, and as a regulatory RNA, controls transcription of 147 genes, including housekeeping and toxin genes, in *C. perfringens*[32,44]. Obana et al. [45] showed that VR-RNA regulates the stability of *colA* mRNA by cleaving the transcript. The processed shorter *colA* transcript was more stable than the longer intact *colA* transcript. It is possible that among other factors, downregulation of *vrr* in 13124^R^ (−158) may have contributed to a decrease in the level of transcription of genes. The *vrr* in NCTR^R^ was upregulated twofold. *virX* is another regulatory gene that, even in the absence of the *VirR/VirS* regulatory system, activates the transcription of the *pfoA*, *plc* and *colA* genes, and its overexpression results in the increased expression of toxin genes [44,46]. qRT-PCR results showed that the expression of this gene increased at least 2.2 times in NCTR^R^ and decreased by −3.0 in 13124^R^.

Another regulatory gene whose expression was altered in the mutants was *revR,* which was downregulated in 13124^R^ and upregulated in NCTR^R^. *revR* is a response regulator that alters the transcription of 100 genes, including those for potential virulence factors, which also are regulated by (VirR/VirS), and those for cell wall metabolism [47]. Hiscox et al. [47] found that a *revR* mutant of *C. perfringens* 13 was filamented. Gram staining of the wild types and mutants of ATCC 13124 and NCTR showed that cells of both mutants were filamented and longer than those of the wild types. Microarray and qRT-PCR analysis (Table 1) showed that some putative membrane protein genes were differentially expressed in the mutants and wild types of both strains.

The amino acid sequences of the toxin genes and the regulatory genes (*virR/virS*) in the mutants and wild types of both strains were identical, except that there were two silent mutations in *virR/virS* in NCTR^R^, so the expression of toxin genes and their regulators was not the result of gene mutation. The sequence of *vrr* was identical in the mutants and wild types of both strains, and the sequence of *revR* in ATCC 13124 and 13124^R^ was also identical. Obana and Nakamura [48] also detected other regulatory genes, CPE_1446-CPE_1447, which appear to regulate the transcription of *plc, pfoA, nanI* and *nagHIJK* at transcription level. Microarray analysis showed that CPE_1447 was downregulated in NCTR^R^, but this gene was not detected in the microarray data from ATCC 13124. qRT-PCR confirmed that *nanI* was downregulated and sialidase was decreased in NCTR^R^; however, the role of CPE_1447 in the regulation of this gene is not clear.

Another global regulatory protein, CodY, has been shown to regulate expression of many genes in *Bacillus subtilis* and *Clostridium difficile*[49,50]. It appears to repress genes whose products are not needed during growth in high nutrient medium. qRT-PCR showed that CodY was upregulated (6.9 times) in NCTR^R^ and downregulated (−1.89 times) in 13124^R^. The sequence of *codY* was identical in both ATCC 13124 and 13124^R^. Since both mutants and wild types were grown in a rich medium, the effect of CodY on alteration of gene expression in our strains is not known.

In addition, microarray analysis also detected some regulatory genes that were downregulated in both mutants (Table 3) and some that were upregulated in NCTR^R^ and downregulated in 13124^R^ (Table 1). Among those genes that were affected differently was CPF_0069, which is a transcription antiterminator similar to the BglG-type regulators in other bacteria (http://www.ncbi.nlm.nih.gov/). This gene was downregulated in 13124^R^ and upregulated in NCTR^R^. At this point, the roles that this gene and others play in altering the transcription of toxin genes in resistant strains are not known. Nor is there a reason known for the contradictory effects of fluoroquinolone resistance selection on the expression of regulatory genes, including those that regulate toxin production, and it needs to be investigated further. Autoinducers (AI-2) also have been implicated in the regulation of some toxin genes [51]. However, in our strains, the production of AI-2 per cell unit, measured by the indicator *Vibrio harveyi*, was higher for 13124^R^ than for ATCC 13124 and lower for NCTR^R^ than for NCTR. The ratio of AI-2 production per OD unit in an overnight culture of the mutant to that of the wild type was 1.5 for ATCC 13124 and 0.14 for NCTR. The contradictory results observed in the transcription of various toxin genes in two resistant strains were accompanied by changes in the levels of toxins and other enzymes. The most dramatic changes were observed for phospholipase C (PLC) and perfringolysin O (PFO). These two toxins were substantially decreased in 13124^R^ and increased in NCTR^R^. The alterations in the production of enzymes were accompanied by changes in cytotoxicity for macrophages. The cytotoxicities of cell-free culture supernatants of the wild type ATCC 13124 and NCTR, for the macrophages were comparable. However, the cell-free culture supernatant of 13124^R^ exhibited significantly lower cytotoxicity for macrophages than ATCC 13124, but that of NCTR^R^ had higher cytotoxicity than NCTR. These data were consistent with the alterations in the transcription patterns of toxin genes and enzyme assays that were observed by DNA microarray analysis, qRT-PCR assay and toxin production. The cytotoxic effects were correlated with the transcription pattern of toxins and virulence-associated genes and enzymatic activities, confirming that the effect of fluoroquinolones on *C. perfringens* was strain-specific. O’Brien and Melville [33] reported that perfringolysin O (PFO) plays a more prominent role than α-toxin (PLC) in cytotoxicity for macrophages. Since we used the crude extract, which contains various factors including PFO and PLC, our results only show the alteration in the overall cytotoxicities of the mutants in comparison with their wild types and the contributing factors and their affinities for macrophage receptors are not known. Fluoroquinolone resistance selection decreased the toxicity of 13124^R^ and increased the toxicity of NCTR^R^.

## Conclusions

Our study demonstrates that gatifloxacin resistance selection in *C. perfringens* was associated with upregulation or downregulation of different genes involved in various aspects of metabolism and that the effect was strain-specific. The genes involved in transcription regulation, virulence and cell toxicity were among those that were upregulated in one resistant strain and downregulated in another. Hiscox et al. [47] surmised that “the regulation of virulence in *C. perfringens* was a complex process” and we found that the nature of each strain adds yet another level of complexity to gene regulation in *C. perfringens*. Myer et al. [52] found widely variable large genomic islands in a large collection of *C. perfringens* strains and stated that considerable variation exists among the genomes of *C. perfringens* strains. It appears that this variation in gene structure of different *C. perfringens* strains also affects gene regulation and interaction of bacteria with fluoroquinolones. Fluoroquinolones have been implied to have a role in the development of *C. difficile* associated diarrhea [53]. Since virulent, drug-resistant clinical isolates of pathogenic bacteria have an undefined genetic basis for their resistance and virulence, we used two wild types and otherwise isogenic resistant mutants, which are difficult to obtain in a clinical setting, to assess fluoroquinolone effects. Our results reflect clinical observations of finding fluoroquinolone-resistant strains of bacteria that are more or less virulent than the susceptible strains. They underscore the role of fluoroquinolones in changing bacterial virulence and the importance of prudent use of fluoroquinolones. Further study is needed on the effect of fluoroquinolones on a larger number of *C. perfringens* strains, along with genomic analysis of the resistant mutants.

## Abbreviations

PLC: Phospholipase C; PFO: Perfringolysin O; gyrA and gyrB: Gyrase; parC and parE: Topoisomerase IV; BHI: Brain Heart Infusion; VirS, VirR, virX, vrr(VR-RNA), Cody RevR: Regulatory genes.

## Competing interests

The authors declare that they have no competing interests.

## Authors’ contributions

Technical experiments and statistical analysis were performed by MP and SP. SP performed those on RT-PCR and cytotoxicity, morphological analysis and MP performed the rest of the experiments. SP wrote the first draft of the manuscript sections on RT-PCR analysis, cytotoxicity and cell morphology. FR planned the experiments, analyzed the data, and wrote the manuscript. All authors have read and approved the final manuscript.

## Supplementary Material

Additional file 1 Primers used for qRT-PCR. Click here for file

Additional file 2 Analysis of mRNA quality and expression. Click here for file

Additional file 3 **Cytotoxicities of *C. perfringens* supernatants for macrophages.** Click here for file

Additional file 4 **Morphological examination of *C. perfringens *strains.** Click here for file

## References

[B1] Scallan E Hoekstra RM Angulo FJ Tauxe RV Widdowson MA Roy SL **Foodborne illness acquired in the United States—major pathogen**s Emerg Infect Dis 2011 17 7 15 21192848 10.3201/eid1701.P11101 PMC3375761

[B2] Heimesaat MM Granzow K Leidinger H Liesenfeld O Prevalence of *Clostridium difficile* toxins A and B and *Clostridium perfringens* enterotoxin A in stool samples of patients with antibiotic-associated diarrhea Infection 2005 33 340 344 10.1007/s15010-005-5067-3 16258864

[B3] Meyns E Vermeersch N Ilsen B Hoste W Delooz H Hubloue I Spontaneous intrahepatic gas gangrene and fatal septic shock Acta Chir Belg 2009 109 400 404 19943601 10.1080/00015458.2009.11680447

[B4] Rood JI Virulence genes of *Clostridium perfringens* Annu Rev Microbiol 1998 52 333 360 10.1146/annurev.micro.52.1.333 9891801

[B5] Sparks SG Carman RJ Sarker MR McClane BA Genotyping of enterotoxigenic *Clostridium perfringens* fecal isolates associated with antibiotic-associated diarrhea and food poisoning in North America J Clin Microbiol 2001 39 883 888 10.1128/JCM.39.3.883-888.2001 11230399 PMC87845

[B6] Petit L Gibert M Popoff MR *Clostridium perfringens:* toxinotype and genotype Trends Microbiol 1997 179 104 110 10.1016/s0966-842x(98)01430-9 10203838

[B7] Titball RW Hunter SE Martin KL Morris BC Shuttleworth AD Rubidge T Molecular cloning and nucleotide sequence of the alpha-toxin (phospholipase C) of *Clostridium perfringens* Infect Immun 1989 57 367 376 2536355 10.1128/iai.57.2.367-376.1989 PMC313106

[B8] Lazarescu C Kimmoun A Blatt A Bastien C Levy B *Clostridium perfringens* gangrenous cystitis with septic shock and bone marrow necrosis Intensive Care Med 2012 38 1906 1907 10.1007/s00134-012-2647-4 22797355

[B9] Gosselink MP Schouten WR van Lieshout LM Hop WC Laman JD Eradication of pathogenic bacteria and restoration of normal pouch flora: comparison of metronidazole and ciprofloxacin in the treatment of pouchitis Dis Colon Rectum 2004 47 1519 1525 10.1007/s10350-004-0623-y 15486751

[B10] Gionchetti P Rizzello F Venturi A Ugolini F Rossi M Brigidi P Antibiotic combination therapy in patients with chronic, treatment-resistant pouchitis Aliment Pharmacol Ther 1999 13 713 718 10.1046/j.1365-2036.1999.00553.x 10383499

[B11] Leal J Gregson DB Ross T Church DL Laupland KB Epidemiology of *Clostridium* species bacteremia in Calgary, Canada, 2000–2006 J Infect 2008 5 198 203 18672296 10.1016/j.jinf.2008.06.018

[B12] Wexler HM Molitoris E Finegold SM In vitro activities of three of the newer quinolones against anaerobic bacteria Antimicrob Agents Chemother 1992 36 239 234 10.1128/AAC.36.1.239 1317149 PMC189287

[B13] Ferrero L Cameron B Crouzet J Analysis of gyrA and grlA mutations in stepwise-selected ciprofloxacin-resistant mutants of *Staphylococcus aureus* Antimicrob Agents Chemother 1995 39 1554 1558 10.1128/AAC.39.7.1554 7492103 PMC162780

[B14] Tamayo M Santiso R Gosalvez J Bou G Fernandez JL Rapid assessment of the effect of ciprofloxacin on chromosomal DNA from *Escherichia coli* using an in situ DNA fragmentation assay BMC Microbiol 2009 9 69 10.1186/1471-2180-9-69 19364397 PMC2670838

[B15] Cirz RT Jones MB Gingles NA Minogue TD Jarrahi B Peterson SN Complete and SOS-mediated response of *Staphylococcus aureus* to the antibiotic ciprofloxacin J Bacteriol 2007 189 531 539 10.1128/JB.01464-06 17085555 PMC1797410

[B16] Dorr T Lewis K Vulic M SOS response induces persistence to fluoroquinolones in *Escherichia coli* PLoS Genet 2009 5 e1000760 10.1371/journal.pgen.1000760 20011100 PMC2780357

[B17] Dorr T Vulic M Lewis K **Ciprofloxacin causes persister formation by inducing the TisB toxin in *****Escherichia col****i* PLoS Biol 2010 8 e1000317 10.1371/journal.pbio.1000317 20186264 PMC2826370

[B18] Dal Sasso M Culici M Bovio C Braga PC Gemifloxacin: effects of sub-inhibitory concentrations on various factors affecting bacterial virulence Int J Antimicrob Agents 2003 21 325 333 10.1016/S0924-8579(02)00391-6 12672578

[B19] Dorman CJ Ni Bhriain N Higgins CF DNA supercoiling and environmental regulation of virulence gene expression in *Shigella flexneri* Nature 1990 344 789 792 10.1038/344789a0 2184366

[B20] Mesak LR Davies J Phenotypic changes in ciprofloxacin-resistant *Staphylococcus aureus* Res Microbiol 2009 160 785 791 10.1016/j.resmic.2009.09.013 19818400

[B21] Muto CA Pokrywka M Shutt K Mendelsohn AB Nouri K Posey K A large outbreak of *Clostridium difficile*-associated disease with an unexpected proportion of deaths and colectomies at a teaching hospital following increased fluoroquinolone use Infect Control Hosp Epidemiol 2005 26 273 280 10.1086/502539 15796280

[B22] Noren T *Clostridium difficile* and the disease it causes Methods Mol Biol 2010 646 9 35 10.1007/978-1-60327-365-7_2 20597000

[B23] Pawlowski SW Archbald-Pannone L Carman RJ Alcantara-Warren C Lyerly D Genheimer CW Elevated levels of intestinal inflammation in *Clostridium difficile* infection associated with fluoroquinolone-resistant *C. difficile* J Hosp Infect 2009 73 185 187 10.1016/j.jhin.2009.05.013 19709778 PMC2743747

[B24] Saxton K Baines SD Freeman J O'Connor R Wilcox MH Effects of exposure of *Clostridium difficile* PCR ribotypes 027 and 001 to fluoroquinolones in a human gut model Antimicrob Agents Chemother 2009 53 412 420 10.1128/AAC.00306-08 18710908 PMC2630646

[B25] Uchida Y Mochimaru T Morokuma Y Kiyosuke M Fujise M Eto F Clonal spread in Eastern Asia of ciprofloxacin-resistant *Escherichia coli* serogroup O25 strains, and associated virulence factors Int J Antimicrob Agents 2010 35 444 450 10.1016/j.ijantimicag.2009.12.012 20188525

[B26] Drews SJ Poutanen SM Mazzulli T McGeer AJ Sarabia A Pong-Porter S Decreased prevalence of virulence factors among ciprofloxacin-resistant uropathogenic *Escherichia coli* isolates J Clin Microbiol 2005 43 4218 4220 10.1128/JCM.43.8.4218-4220.2005 16081983 PMC1233890

[B27] Ferjani S Saidani M Ennigrou S Hsairi M Ben Redjeb S Virulence determinants, phylogenetic groups and fluoroquinolone resistance in *Escherichia coli* isolated from cystitis and pyelonephritis Pathol Biol (Paris) 2012 60 270 274 10.1016/j.patbio.2011.07.006 21872408

[B28] Sun J Hu J Peng H Shi J Dong Z Molecular and physiological characterization of fluoroquinolone resistance in relation to uropathogenicity among *Escherichia coli* isolates isolated from Wenyu River, China Chemosphere 2012 87 37 42 10.1016/j.chemosphere.2011.11.050 22182707

[B29] Rafii F Park M Novak JS Alterations in DNA gyrase and topoisomerase IV in resistant mutants of *Clostridium perfringens* found after in vitro treatment with fluoroquinolones Antimicrob Agents Chemother 2005 49 488 492 10.1128/AAC.49.2.488-492.2005 15673722 PMC547304

[B30] Rafii F Park M Bryant AE Johnson SJ Wagner RD Enhanced production of phospholipase C and perfringolysin O (alpha and theta toxins) in a gatifloxacin-resistant strain of *Clostridium perfringens* Antimicrob Agents Chemother 2008 52 895 900 10.1128/AAC.01316-07 18160514 PMC2258526

[B31] Rafii F Park M Gamboa da Costa G Camacho L Comparison of the metabolic activities of four wild-type *Clostridium perfringens* strains with their gatifloxacin-selected resistant mutants Arch Microbiol 2009 191 895 902 10.1007/s00203-009-0518-3 19855959 PMC12786728

[B32] Ohtani K Hirakawa H Tashiro K Yoshizawa S Kuhara S Shimizu T Identification of a two-component VirR/VirS regulon in *Clostridium perfringens* Anaerobe 2010 16 258 264 10.1016/j.anaerobe.2009.10.003 19835966

[B33] O'Brien DK Melville SB Effects of *Clostridium perfringens* alpha-toxin (PLC) and perfringolysin O (PFO) on cytotoxicity to macrophages, on escape from the phagosomes of macrophages, and on persistence of *C. perfringens* in host tissues Infect Immun 2004 72 5204 5215 10.1128/IAI.72.9.5204-5215.2004 15322015 PMC517428

[B34] Awad MM Ellemor DM Bryant AE Matsushita O Boyd RL Stevens DL Construction and virulence testing of a collagenase mutant of *Clostridium perfringens* Microb Pathog 2000 28 107 117 10.1006/mpat.1999.0328 10644496

[B35] Dargatz H Diefenthal T Witte V Reipen G von Wettstein D The heterodimeric protease clostripain from *Clostridium histolyticum* is encoded by a single gene Mol Gen Genet 1993 240 140 145 8341259 10.1007/BF00276893

[B36] Li J Sayeed S Robertson S Chen J McClane BA Sialidases affect the host cell adherence and epsilon toxin-induced cytotoxicity of *Clostridium perfringens* type D strain CN3718 PLoS Pathog 2011 7 e1002429 10.1371/journal.ppat.1002429 22174687 PMC3234242

[B37] Song JM Im JH Hoon JH Kang JD Kang DJ A simple method for hyaluronic acid quantification in culture broth Carbohydr Polym 2009 78 633 634 10.1016/j.carbpol.2009.04.033

[B38] Kugelberg E Lofmark S Wretlind B Andersson DI Reduction of the fitness burden of quinolone resistance in *Pseudomonas aeruginosa* J Antimicrob Chemother 2005 55 22 30 15574475 10.1093/jac/dkh505

[B39] Marcusson LL Frimodt-Moller N Hughes D Interplay in the selection of fluoroquinolone resistance and bacterial fitness PLoS Pathog 2009 5 e1000541 10.1371/journal.ppat.1000541 19662169 PMC2714960

[B40] Bachoual R Tankovic J Soussy CJ Analysis of the mutations involved in fluoroquinolone resistance of in vivo and in vitro mutants of *Escherichia coli* Microb Drug Resist 1998 4 271 276 10.1089/mdr.1998.4.271 9988045

[B41] Smani Y Lopez-Rojas R Dominguez-Herrera J Docobo-Perez F Marti S Vila J In vitro and in vivo reduced fitness and virulence in ciprofloxacin-resistant ***Acinetobacter baumannii.*** Clin Microbiol Infect 2012 18 1 4 10.1111/j.1469-0691.2011.03695.x 22084991

[B42] Shimizu T Shima K Yoshino K Yonezawa K Hayashi H P**roteome and transcriptome analysis of the virulence genes regulated by the VirR/VirS system in *****Clostridium perfringens*** J Bacteriol 2002 184 2587 2594 10.1128/JB.184.10.2587-2594.2002 11976286 PMC135029

[B43] Shimizu T Yaguchi H Ohtani K Banu S Hayashi H Clostridial VirR/VirS regulon involves a regulatory RNA molecule for expression of toxins Mol Microbiol 2002 43 257 265 10.1046/j.1365-2958.2002.02743.x 11849553

[B44] Okumura K Ohtani K Hayashi H Shimizu T Characterization of genes regulated directly by the VirR/VirS system in *Clostridium perfringens* J Bacteriol 2008 190 7719 7727 10.1128/JB.01573-07 18790863 PMC2583603

[B45] Obana N Shirahama Y Abe K Nakamura K Stabilization of *Clostridium perfringens* collagenase mRNA by VR-RNA-dependent cleavage in 5' leader sequence Mol Microbiol 2010 77 1416 1428 10.1111/j.1365-2958.2010.07258.x 20572941

[B46] Ohtani K Bhowmik SK Hayashi H Shimizu T Identification of a novel locus that regulates expression of toxin genes in *Clostridium perfringens* FEMS Microbiol Lett 2002 209 113 118 10.1111/j.1574-6968.2002.tb11118.x 12007663

[B47] Hiscox TJ Chakravorty A Choo JM Ohtani K Shimizu T Cheung JK Regulation of virulence by the RevR response regulator in *Clostridium perfringens* Infect Immun 2011 79 2145 2153 10.1128/IAI.00060-11 21402758 PMC3125849

[B48] Obana N Nakamura K A novel toxin regulator, the CPE1446-CPE1447 protein heteromeric complex, controls toxin genes in *Clostridium perfringens* J Bacteriol 2011 193 4417 4424 10.1128/JB.00262-11 21725013 PMC3165525

[B49] Brinsmade SR Sonenshein AL Dissecting complex metabolic integration provides direct genetic evidence for CodY activation by guanine nucleotides J Bacteriol 2011 193 5637 5648 10.1128/JB.05510-11 21856856 PMC3187194

[B50] Dineen SS McBride SM Sonenshein AL Integration of metabolism and virulence by *Clostridium difficile* CodY J Bacteriol 2010 192 5350 5362 10.1128/JB.00341-10 20709897 PMC2950512

[B51] Ohtani K Yuan Y Hassan S Wang R Wang Y Shimizu T Virulence gene regulation by the agr system in *Clostridium perfringens* J Bacteriol 2009 191 3919 3927 10.1128/JB.01455-08 19363118 PMC2698399

[B52] Myers GS Rasko DA Cheung JK Ravel J Seshadri R DeBoy RT Skewed genomic variability in strains of the toxigenic bacterial pathogen, *Clostridium perfringens* Genome Res 2006 16 1031 1040 10.1101/gr.5238106 16825665 PMC1524862

[B53] Deshpande A Pant C Jain A Fraser TG Rolston DD Do fluoroquinolones predispose patients to *Clostridium difficile* associated disease? A review of the evidence Curr Med Res Opin 2008 24 329 333 10.1185/030079908X253735 18067688

