# Supplementary material for: Comparative transcription analysis and toxin production of two fluoroquinolone-resistant mutants of Clostridium perfringens
Source: BMC Microbiol. 2013 Mar 1;13:50. doi: 10.1186/1471-2180-13-50 (PMC3599539; doi:10.1186/1471-2180-13-50)
Supplement: Additional file 1 — Primers used for qRT-PCR. [file 1471-2180-13-50-S1.pdf]

Additional file 1.
Primers used for qRT-PCR

**Cell envelope**	
CPF_1345	AGAGGGAATTTAAAGCTTAATGA<br>TTTTTATATAGATGATATGATGCCTGT
CPF_0155 (*pfoR*)	TAAGGCACCACATGGAATGA<br>ACTCCTCCCAAGCTTCCATT
CPF_0244	TCCAGGAAAAGCAGGAATTG<br>GCAAATTCCCTAACAACGTCA
CPF_0274	CGCACCTAACAGAGGTGGAG<br>TGGACCAGTTGCACCAAATA
CPF_0710	GCACCATTTTGGGAAGAAAC<br>TGAGCCTGCTCCCATACTTC
**Cellular processes**	
CPF_0042 (*plc*)	TGACACAGGGGAATCACAAA<br>CGCTATCAACGGCAGTAACA
CPF_0840 (*cloS1*)	TGGTGGGGCTAAGGATGATA<br>CCAAACAACTGGTGCTGATG
CPF_1725 (*hlyC*)	CGGGATTGGATTACCACTTTT<br>AAGGCATATAACTTCCGCAGAT
CPF_0156 (*pfoA*)	TGGAGCCTATGTTGCACAGT<br>CCAAGCAAGGCCTGTACACT
CPF_0784 (*ahpC*)	TGGGCAGATGCTTCAGATACT<br>TGCAACAAACTGAGCTGCTT
CPF_1348 (*pac*)	TAATGAAAAGGGGCTTGCT<br>TTCCAAGCTCTCCTAATGAAGG
**Energy metabolism**	
CPF_0780	TGACACAGGGGAATCACAAA<br>CGCTATCAACGGCAGTAACA
CPF_1505 (*eno*)	GCAGGTTATGAGCCAGGAAA<br>TCTTCGGCCATACCATCTTC
CPF_2315	CAGGTGGTATGTTTGCTCCA<br>TGCCATTGTTGGTATTTTTCC
CPF_2747	CTCTTGTCCATGGTGCGTTA<br>AGCATTTTTATCGAAACCAACT
CPF_2875	GCTTTATTCTGCCCAACAGG<br>CCTGCTGCGTTTACTACGAA
**Purines, pyrimidines, nucleotides, and nucleosides**	
CPF_2558 (*guaB*)	AGCAGAAGCAGGTGCAGATT<br>TGCTTCTTCACATCCTGCAA
CPF_2958 (*purA*)	TGAAGGAGCTCAAGGAATGC<br>CAAGCTCTGTTGGGAATGGT
**Protein fate**	
CPF_0166 (*colA*)	TAGGAACAAAGGCGCAAGAT

	TAGGAACAAAGGCGCAAGAT
CPF_2632	GTGGGAATCCAACTCCAAAG<br>TCTACATCCCCAGCTGTTCC
CPF_1002	TGCCAATGTAATCCCTGACA<br>TAAGGTTGGGCATCCACTTC
**Regulatory functions**	
CPF_0069	ATTCGGCAAGAACAACAGGA<br>GCAACCTTAAAGGATTCTGGA
CPF_0753	TCAAATTGGCTTTATGGAGGA<br>GGGAAGAGCCTCATTATTTGC
CPF_1784 (*scrR*)	ATATCCTCCAAAGGCTGCAA<br>ATATCCTCCAAAGGCTGCAA
CPF_2292 (*hrcA*)	TGCCAATGTAATCCCTGACA<br>TAAGGTTGGGCATCCACTTC
CPF_2673	GCCAGAGGAATGCATGGTTA<br>TCATCTCCTCTTGCAAATCTTTC
CPF_1955 (*codY*)	TGTGTTTTTGAAGGGGTAGGA<br>GCTAATTGAACCACCGCTTT
CPF_1752 (*virR*)	TGGGTGAGTTAACAGGAATGG<br>CACAAGCTGTAACCTGCTCTTG
CPF_1751 (*virS*)	TTTAAAGGGGGACGAGAAGG<br>TTCCTTCAATACAGGCTATGTGAT
CPF_0627 (*virX*)	TGGAAAAGAATTCGTATTCACTGTA<br>TCTTGCTTTTCTGCAAGCTG
CPF_1204 (*vrr*)	CAAAAAGGATTTTAACAAGTGCAA<br>TTGATATTAAAGCAAGTATGGGACT
CPE0845 (*virT*)	TCATTAGGCAGGAGCTTTTCA<br>TGATATTGCCACCCCAACTT
**Transport and binding proteins**	
CPF_1450 (*mgtE*)	GGTGGAAATGGAGGAACTCA<br>TTCCAATTGCAGGTTTTCCT
CPF_1507	TTGGATTTTTCGCAATGACA<br>ACTCCACCTTTTTCCCATCC
CPF_1756 (*uraA*)	CAGAGCATGTTGGCCATTTA<br>CAGATAAAACCCCAGCTCCA
CPF_0070	GCTCCTGCAACTGGAAAAAC<br>AAAGTCATAGCGAAAGCATGG
CPF_0703	TGCAGTAATGCATGATCCAG<br>CCACATCAGCCATTCTGTGA
CPF_0756 (*gltT*)	CCACTTGGCGTATTTGGATT<br>ACGGCTTCAGAACTTGCTGT
CPF_1621	ATGGATGCGGTATGGTTGTT<br>TGGCCCAGATGAAGGATTTA
CPF_2341 (*modB*)	AAATGCTGCTAGGGAAATGG<br>TTTGCTAAACCTGAATCACCA
CPF_2652	GAACATTCCAAAAGGCTGGA<br>CTGCTCCTTCTTCTGCAACC
**Unknown functions**	

CPF_0176	GGAGATGATTGCAGAAACAGC<br>CACTTCCTGCCTTTGTTAAAGC
CPF_1375	GAACAAAAAGGGGATAGGGTTT<br>TTTGCTTTCAGAATCCCACA
CPF_2038 (*nifU*)	CGGAATCGGAGAAGTTGGT<br>GCTTCTGCAACAGCTTTGTTT
CPF_2758	CTTGGACCATGTGGAAGACC<br>TTTCTTCATATGTGCTTTGCTCA
