# Supplementary material for: Comparative transcription analysis and toxin production of two fluoroquinolone-resistant mutants of Clostridium perfringens
Source: BMC Microbiol. 2013 Mar 1;13:50. doi: 10.1186/1471-2180-13-50 (PMC3599539; doi:10.1186/1471-2180-13-50)
Supplement: Additional file 2 — Analysis of mRNA quality and expression. [file 1471-2180-13-50-S2.pdf]

Additional file 2. **Analysis of mRNA quality and expression**

All RNA had an A260/280 ratio greater than 2, indicating very little or no protein contamination. RNA integrity analysis was performed for RNA that was used for microarray experiments by using an Agilent 2100 bioanalyzer. All rRNA ratios (23S/16S) were above 1.4 (range 1.4-1.9). The total RNA is considered intact for mRNA enrichment if the rRNA ratio is equal to or greater than 1. During qRT-PCR analysis, melting curves for each gene amplified were monitored.

The mean expression levels of the genes from the wild types and the mutants were calculated by qRT-PCR from their mRNA. The $2^{-\Delta\Delta C}{}_T$ (Livak) method, as described in the Bio Rad real time application guide, was used to calculate the relative change in gene expression. Cycle threshold (C_T), number of PCR cycles for each gene for the mutants and wild types, and

16S rRNA as internal control (for normalization) were determined. A lower C_T represents a higher amount of target, and vice versa. In each experiment, the ΔC_T values for the mutants and wild type genes were determined.

ΔC_{Tm} = (C_T of mutant genes - C_T of 16S $rRNA_m$).

ΔC_{Tw} = (C_T of wild type gene - C_T of 16S rRNA $_w$).

$\Delta\Delta C_T$ = (ΔC_T of mutant - ΔC_T of wild type).

Due to the exponential nature of PCR, the relative change was converted to a linear form $2^{-\Delta\Delta C}{}_T$.

Fold increase in mRNA expression of the mutant was defined as N-fold $\geq$1.5 and decrease in mRNA expression was $\leq$0.6 or -1.5.

Livak, KJ, Schmittgen,TD: **Analysis of relative gene expression data using real-time quantitative PCR and the $2^{-\Delta\Delta}C_T$ method**. *METHODS* 2001 **25:**402–408.
