# Supplementary material for: Comparative transcription analysis and toxin production of two fluoroquinolone-resistant mutants of Clostridium perfringens
Source: BMC Microbiol. 2013 Mar 1;13:50. doi: 10.1186/1471-2180-13-50 (PMC3599539; doi:10.1186/1471-2180-13-50)
Supplement: Additional file 3 — Cytotoxicities of C. perfringens supernatants for macrophages. [file 1471-2180-13-50-S3.pdf]

Additional file 3: Cytotoxicities of *C. perfringens* supernatants for macrophages

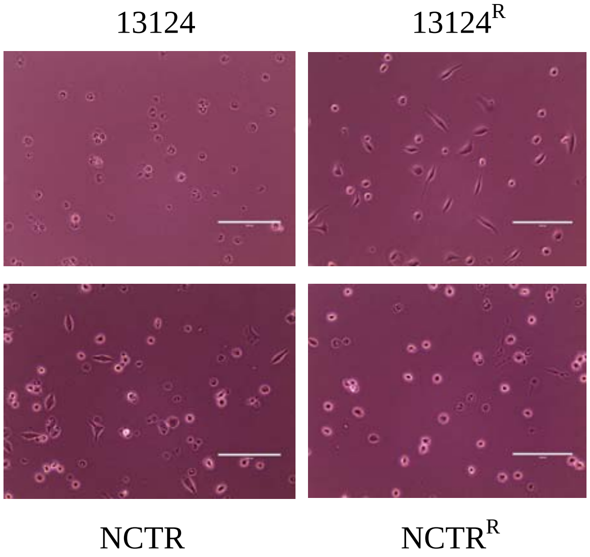

Representative microscopic images of macrophages taken with an inverted microscope after 18 h of incubation with cell-free filtrates of *C. perfringens* wild type and gatifloxacin resistant mutants.
