# Supplementary material for: Comparative transcription analysis and toxin production of two fluoroquinolone-resistant mutants of Clostridium perfringens
Source: BMC Microbiol. 2013 Mar 1;13:50. doi: 10.1186/1471-2180-13-50 (PMC3599539; doi:10.1186/1471-2180-13-50)
Supplement: Additional file 4 — Morphological examination of C. perfringens strains. [file 1471-2180-13-50-S4.pdf]

Additional file 4. Morphological examination of *C. perfringens* strains

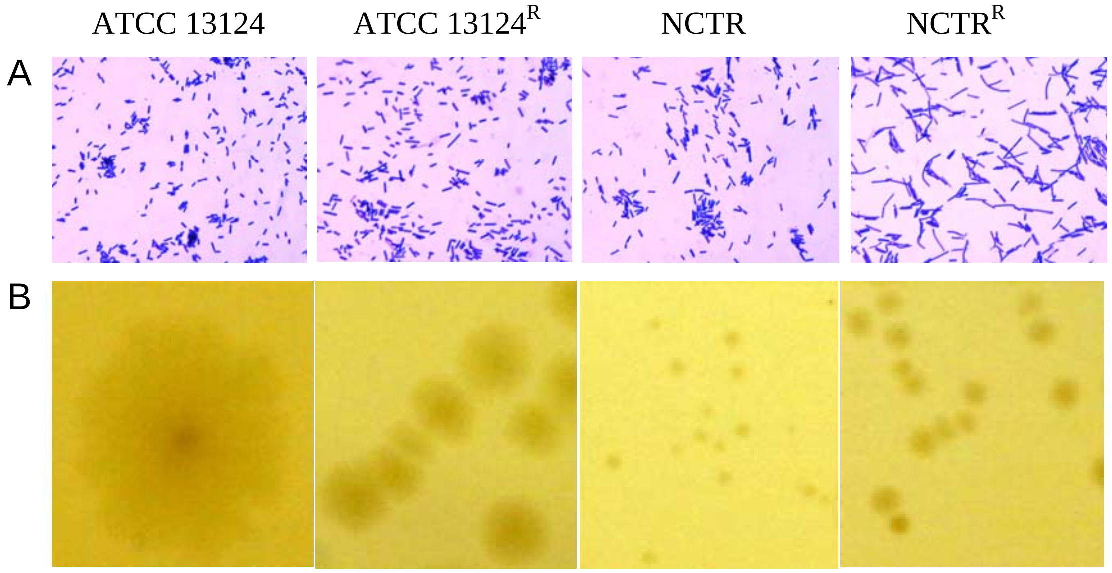

Comparison of gatifloxacin-resistant *C. perfringens* mutant strains (ATCC 13124^R and $NCTR^R$) with their parental wild type strains ATCC 13124 and NCTR. **A**: Gram staining (mag. 1000x). The resistant strains were elongated, but the elongation was more pronounced in the gatifloxacin resistant mutant $NCTR^R$. **B**: Changes in colony size and morphology after gatifloxacin resistance selection.
